# A Systematic Review Evaluating the Diagnostic Efficacy of Narrow-Band Imaging for Laryngeal Cancer Detection

**DOI:** 10.3390/medicina60081205

**Published:** 2024-07-25

**Authors:** Ileana Alexandra Sanda, Razvan Hainarosie, Irina Gabriela Ionita, Catalina Voiosu, Marius Razvan Ristea, Adina Zamfir Chiru Anton

**Affiliations:** 1 “Carol Davila” University of Medicine and Pharmacy, General Medicine, 050474 Bucharest, Romania; ileana-alexandra.sanda@drd.umfcd.ro (I.A.S.); marius-razvan.ristea@drd.umfcd.ro (M.R.R.); 2 ENT Institute of Phonoaudiology and Functional Surgery “Prof. Dr. D. Hociota”, 050751 Bucharest, Romania; 3 Bucharest Emergency University Hospital, 050098 Bucharest, Romania; 4 “Grigore Alexandrescu” Emergency Children Hospital, 011732 Bucharest, Romania

**Keywords:** laryngeal carcinoma, larynx carcinoma, laryngeal cancer, larynx cancer, laryngeal neoplasms, narrow-band imaging, NBI

## Abstract

*Background*: Narrow-band imaging is an advanced endoscopic technology used to detect changes on the laryngeal tissue surface, employing a comparative approach alongside white-light endoscopy to facilitate histopathological examination. *Objective*: This study aimed to assess the utility and advantages of NBI (narrow-band imaging) in identifying malignant laryngeal lesions through a comparative analysis with histopathological examination. *Methods*: We conducted a systematic literature review, utilizing databases such as PubMed, the CNKI database, and Embase for our research. *Results*: We analyzed the articles by reviewing their titles and abstracts, selecting those we considered relevant based on determined criteria; in the final phase, we examined the relevant studies according to the specific eligibility criteria. *Conclusions*: Narrow-band imaging is an advanced endoscopic technology that demonstrates its efficacy as a tool for diagnosing malignant laryngeal lesions and comparing them to premalignant lesions. The European Society of Laryngology has implemented a standardized classification system for laryngeal lesions to enhance data correlation and organization.

## 1. Introduction

Narrow-band imaging (NBI) is an optical endoscopic technology that improves image quality and provides high-resolution sight of the vascular and mucosal tissue network [1].

NBI is a technology designed to improve visualization of the superficial network of capillaries and mucosal vessels [2].

The technology was developed by the Japanese company Olympus (Tokyo, Japan) and Dr. Satoshi Yoshizumi of the University of Tokyo.

The technology works by filtering white light at typical wavelengths—415 nm for blue light and 540 nm for green light—which are absorbed by hemoglobin. As a result, capillaries on the mucosal surface are revealed in shades of brown, while submucosal vessels appear cyan [2].

To optimize the utility of narrow-band imaging (NBI) technology, a standardized classification system for vascular patterns seen under NBI, with a particular focus on intraepithelial capillary loop (IPCL) patterns, is essential for accurate assessment of these lesions.

Therefore, NBI serves as an “optical biopsy”, allowing us to separate malignant from benign lesions, leading to the early characterization of cases even prior to histopathological examination, which remains the gold standard for diagnosing laryngeal malignant lesions [2].

Based on statistical data evidence, laryngeal cancer is the most frequent type of head and neck cancer and represents about 30% of all malignant tumors in this anatomical region [2,3].

Risk factors for laryngeal cancer are multiple and include primarily excessive tobacco and alcohol consumption, gastro-esophageal reflux disease, Plummer–Vinson syndrome, exposure to heat, chemicals, as well as certain viral infections (HPV) [4]. Smoking is considered the most critical risk factor, with a direct link to most cases of laryngeal cancer. Excessive alcohol consumption, especially when combined with smoking, significantly increases the risk of laryngeal cancer [5].

Recent advances in the management of laryngeal cancer have given significant emphasis to preserving laryngeal function, thereby enhancing the overall quality of life for patients.

Narrow-band imaging (NBI) plays a key role in many aspects of laryngeal cancer management. It is a valid tool for early detection, helping to identify tumors. NBI facilitates accurate and efficient tumor resection, contributing to an optimal outcome. In addition, NBI is essential in postoperative follow-up, allowing for prompt detection of local relapses [6,7].

When it comes to the unique advantages of NBI in laryngeal cancer detection, we can confidently state that NBI’s ability to identify precancerous and cancerous lesions of the laryngeal mucosa, which can be challenging to observe with white-light endoscopy (WLE), is a testament to its precision. The use of narrow light filters accentuates vascular and mucosal structures, providing a detailed and specific image that surpasses white-light endoscopy [8]. This precision, coupled with its non-invasiveness and fast, accurate results, instills confidence in its diagnostic capabilities [9,10].

However, it is important to note that NBI comes with its challenges. Implementing NBI necessitates specific, potentially costly equipment, and interpreting NBI images demands training and experience to ensure accurate diagnosis. Furthermore, not all medical centers are equipped with NBI technology, which can limit patient access to this diagnostic method [11].

This study aimed to collect data by conducting a meta-analysis of the literature on the effectiveness and efficacy of NBI in the early detection of laryngeal cancer among patients and their postoperative follow-up.

In the visualization of NBI, different vascular types can be distinguished and classified as follows [12]: In type I, intraepithelial papillary capillary loops are barely visible, while oblique and small-diameter branched vessels are distinctly visible [13]. In type II, intraepithelial papillary capillary loops appear equally thin but with an increased diameter of oblique and branched vessels distinguishable [14]. Type III is characterized by a white mucosal stain that obscures the intraepithelial papillary capillary loops; if the white stain is thin, the oblique and branching vessels may be barely perceptible, but if the white patch is thick, the vessels will be entirely obscured [15]. In type IV, the intraepithelial papillary capillary loops of the mucosa have a visible, relatively regular, low-density arrangement [16,17].

The capillary endings are bifurcated or slightly dilated, presenting as small, scattered, dark brown spots. Oblique and branched vessels are usually not discernible. In type V, changes are further classified into Va, Vb, and Vc based on the vessels’ shape, regularity, and distribution. Lesions seen under NBI are usually classified as A—malignant (type V); B—suspicious for malignancy (bulging or ulcerative lesions covered with necrotic tissue or leukoplakia of unknown type); or C—benign (types I–IV) [17,18].

Like any technique, NBI also has limitations; it does not provide a diagnosis of certainty. This is only achieved with the help of histopathological examination, and management must take into account the patient’s comorbidities, which can be intricate and challenging to diagnose in some cases [19].

In addition to the orientation of laryngeal cancer diagnosis with the help of NBI, there is also white-light fiberscopy, which helps us to visualize tumor formation at the laryngeal level macroscopically, and laryngeal biopsy, which gives us a positive diagnosis of the lesion [20].

Other ‘refined’ endoscopies and techniques include contact video endoscopy, digital filters such as SPIES, and optical filters. These methods, along with NBI endoscopy, contribute to the diverse range of diagnostic tools available for laryngeal cancer detection [21].

## 2. Materials and Methods

### 2.1. Search Methodology

This systematic review was conducted in accordance with the Preferred Reporting Items for Systematic Review and Meta-Analysis (PRISMA) statement to ensure a scientifically robust research approach aimed at lessening bias through the systematic assembly, critical collecting, and synthesis of all significant studies on this topic.

The databases researched included PubMed Clinical Queries, the CNKI database, and Embase. In addition, we meticulously analyzed the reference lists of retrieved articles and cross-referenced them to identify any other relevant articles, all of which were embedded in this analysis.

Our search strategy employed various combinations of the following terms to maximize the yield: “laryngeal carcinoma”, “larynx carcinoma”, “laryngeal cancer”, “larynx cancer”, “laryngeal neoplasms”, “narrow band imaging”, and “NBI”.

### 2.2. Criteria for Eligibility

The inclusion criteria involved original research articles that specifically employed narrow-band imaging (NBI) as a diagnostic tool for examining patients with suspicious laryngeal lesions. Furthermore, eligible studies were required to report on various diagnostic parameters, including sensitivity and specificity, and exhibit transparent specification of the selection criteria. Additionally, studies were limited to those published in English to ensure consistency in language comprehension and data interpretation (Table 1).

The exclusion criteria included studies focusing on lesions in anatomical sites other than the larynx, and reviews, editorials, commentaries, theses, and conference abstracts. In addition, the data extraction process encountered challenges in obtaining complete information; studies that mixed white-light (WL) statistical data with narrow-band imaging (NBI) were also excluded (Table 2).

### 2.3. Information Retrieval Process

Independent reviewers conducted data extraction, capturing details such as the primary author, publication date, geographical origin, article type, patient count, NBI endoscopy system employed, criteria for identifying positive lesions, and number of histopathologically confirmed diagnoses of larynx cancer.

### 2.4. Appraisal of Study Quality

**Figure 1:**
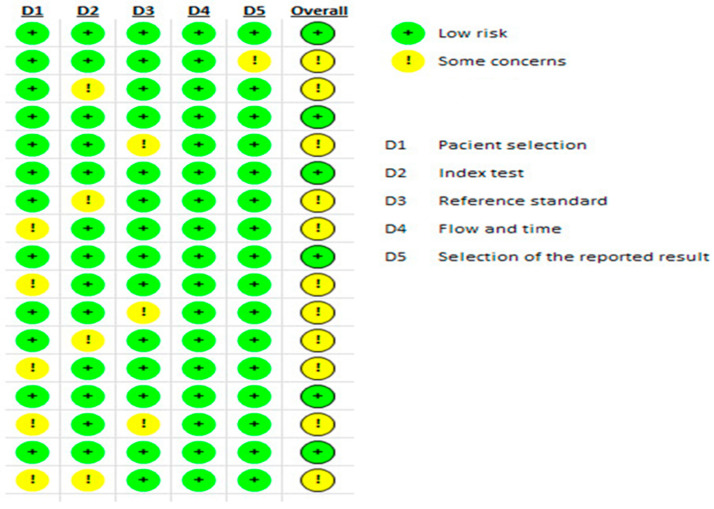
Evaluation of methodological quality using Quality Assessment of Diagnostic Accuracy Studies-2 (QUADAS-2) framework.

Reviewers assessed the methodological quality and potential bias using the Quality Assessment of Diagnostic Accuracy Studies-2 (QUADAS-2). This tool comprises four sections: patient selection, index test, reference standard, and flow and timing. Based on the presence of potential biases, each section contains specific items rated as high risk, low risk, or unclear (Figure 1).

The quality assessment findings of the included articles, as assessed using the QUADAS-2 framework, are detailed herein. Among the 17 studies examined, 6 studies successfully fulfilled all five assessment criteria. Nine studies adhered to four criteria, and one study met three criteria (Figure 1).

**Figure 2:**
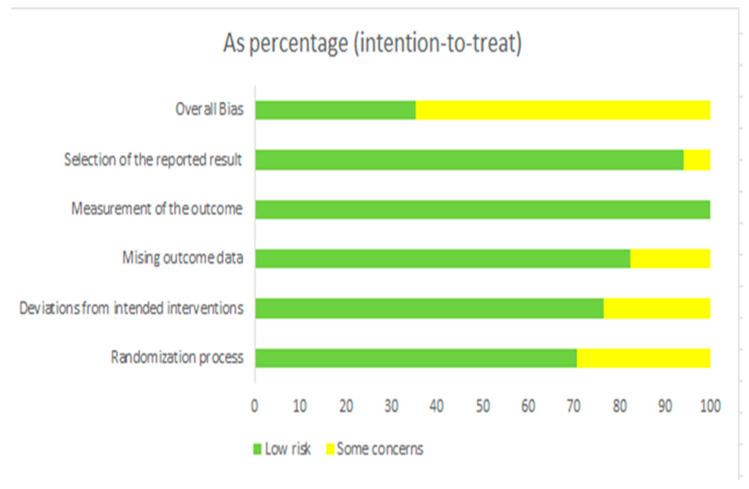
Evaluation of methodological quality using QUADAS-2 framework—percentage.

In Figure 2, the survey assesses bias domains, focusing on the intention-to-treat analysis. Each domain is categorized as “low risk” or “some concern” based on risk levels. Overall, most domains show low risk with minimal concerns. The “Randomization process” has the highest level of concern, with approximately 30% of the data indicating “some concerns”. Similarly, “Missing outcome data” raises about 25% concerns. Other areas such as “Measurement of the outcome”, “Selection of reported results”, and Á “Deviations from intended interventions” demonstrate lower levels of concern, highlighting the study’s overall robustness. Despite some minor issues, the study maintains an overall low risk of bias in most of the areas assessed.

## 3. Results

### 3.1. Included Studies

Following an online search, we initially identified 673 articles with potential impact. Subsequent elimination of duplicates and examination of titles and abstracts resulted in 44 articles being retained for further evaluation. Following an exhaustive review of the complete texts, 18 studies were omitted from consideration as they did not specifically examine the diagnostic efficacy of narrow-band imaging (NBI) in the surveillance of patients previously treated for larynx cancer. Additionally, six studies were excluded due to inadequate data availability, while three studies were disregarded due to their nature as editorial pieces, case reports, or theses.

**Figure 3:**
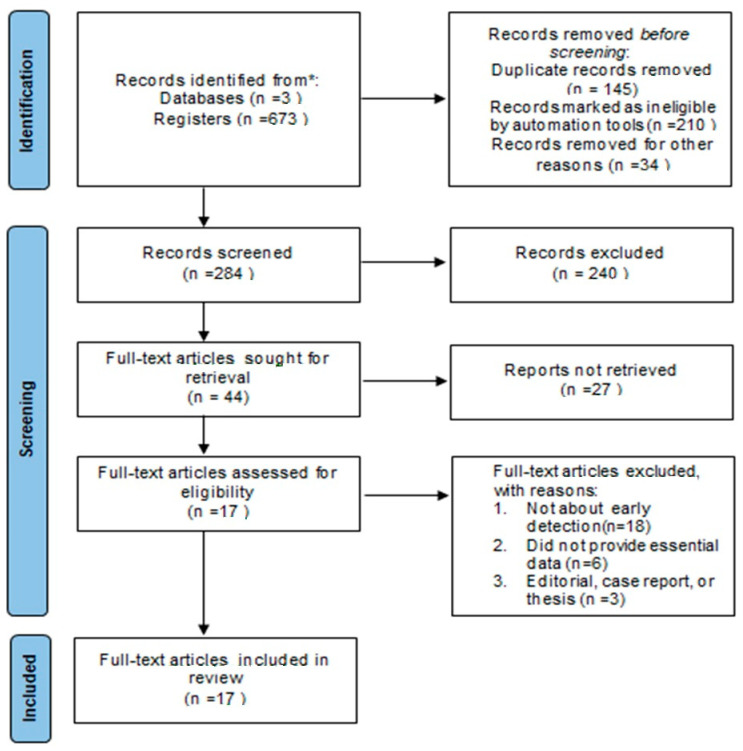
A flow chart of the article selection process.

Ultimately, 17 studies meeting the specified inclusion criteria were chosen for incorporation into the meta-analysis. The procedure for selecting and filtering these studies adhered to the guidelines outlined in the PRISMA (Preferred Reporting Items for Systematic Reviews and Meta-analysis) statement. Figure 3 provides a flowchart illustrating the process of article selection. Table 3 summarizes the essential characteristics of the eligible studies.

### 3.2. Diagnostic Accuracy as Assessed Using Narrow-Band Imaging

**Figure 4:**
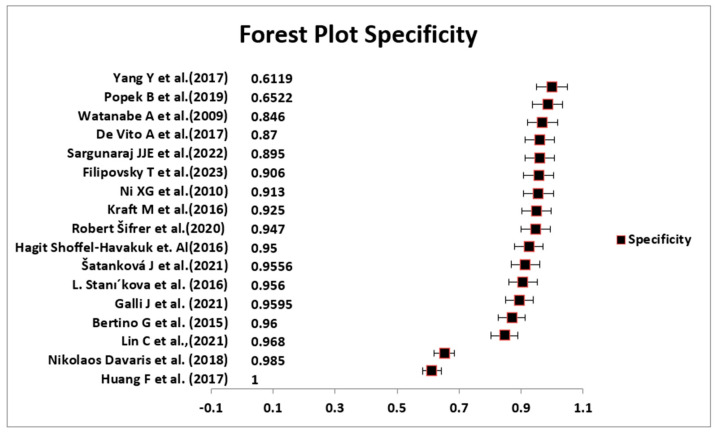
Forest plot for *specificity* of NBI [22,23,24,25,26,27,28,29,30,31,32,33,34,35,36,37,38].

**Figure 5:**
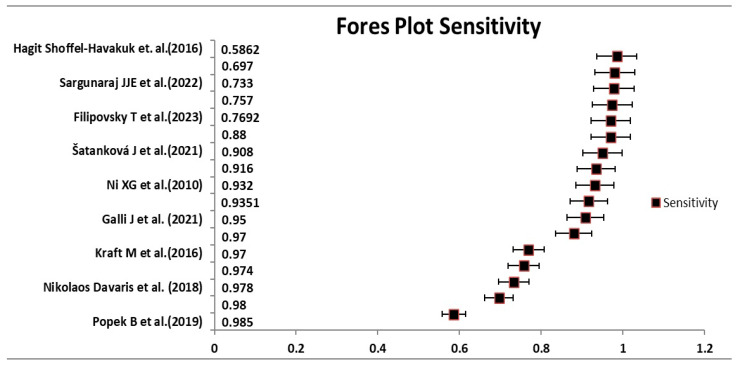
Forest plot for sensitivity of NBI [23,24,25,27,28,29,34,35,37].

In the analysis, the sensitivity and specificity of the 17 included studies are depicted in Figure 4 and Figure 5 through a forest plot and Table 4. The pooled sensitivity and specificity were determined to be 0.87 (95% CI: 0.81–0.94) and 0.90 (95% CI: 0.85–0.96), respectively.

## 4. Discussion

This systematic review is of significant importance as it evaluates the diagnostic efficacy of narrow-band imaging for laryngeal cancer detection, a crucial area of research in the field of laryngeal cancer diagnosis.

The underlying goal of the review analysis is to recognize the opportunities presented by narrow-band imaging (NBI) technology within the framework of optical biopsy-based diagnostics. This involves an examination of the following aspects: an assessment of endoscopic image clarity, the ability to discern and accurately locate the laryngeal lesion, an assessment of cost-effectiveness, and a comparative analysis between clinical suspicion of malignancy and histopathological or immunological validation.

This review aims to evaluate the utility and advantages of narrow-band imaging (NBI) in the diagnosis of laryngeal cancers by assessing its specificity and sensitivity. Based on a review of the current literature, the present study confirms that NBI is an effective diagnostic tool in detecting precancerous and cancerous lesions and identifying suitable surgical margins.

Narrow-band imaging (NBI) is proving valuable in cases where laryngeal lesions are challenging to assess by white-light (WL) examination. Thus, acquiring substantial expertise in NBI interpretation becomes crucial to accurately discern laryngeal lesions, thereby minimizing diagnostic inaccuracies and reducing patient handling times.

Like any technique, it has limitations. These include accessibility and the high costs required for specialized equipment, which limit access to this technology in specific medical centers, whether hospitals or clinics. Medical staff must have experience and training in using this technique. Not all centers are equipped, and misdiagnosis may occur due to a lack of experience. Although NBI improves the visualization of blood vessels in laryngeal structures, its resolution may be insufficient to detect the most minor pathological structures in some cases [10,11].

As a technique that does not require anesthesia, NBI heavily depends on each patient’s anatomic variability and cooperation. Patient engagement is a critical element in ensuring the accurate and thorough assessment of the lesion being followed. This emphasis on patient cooperation underscores the need for their active involvement in the diagnostic process [12].

Interpretation of NBI images may vary between observers, leading to differences in the final diagnosis. Thus, a biopsy is a superior diagnostic method to NBI.

### 4.1. Principal Discoveries and Clinical Implication

In the present study, we included 17 articles and conducted a meta-analysis to assess the efficacy of narrow-band imaging (NBI) in diagnosing laryngeal cancer.

### 4.2. Future Directions

The use of NBI technology offers significant clinical advantages as it saves time, is well received by patients, does not require general anesthesia, and facilitates a thorough examination. In addition to NBI, many other horizontal endoscopic techniques are available, such as autofluorescence, hyperspectral imaging, the SPIES system with the standard version or with Clara, Chroma, A/B spectra filters, i-scan, and AI. Contact endoscopy, in combination with NBI, allows for in vivo and situ examination of lesions, especially in certain situations. We believe NBI should be used as a standard diagnostic tool in patient diagnosis and post-treatment follow-up.

### 4.3. Limitations of the Study

The work included in our study consists of both prospective and retrospective analyses conducted in individual institutions. With a total of 3029 patients included in the studies reviewed, the findings are relatively robust. It is important to note that our access was limited to papers published in English due to resource constraints.

## 5. Conclusions

In conclusion, this meta-analysis highlights the high diagnostic accuracy and efficacy of narrow-band imaging (NBI) in individuals diagnosed with laryngeal cancer (LC), presenting it as a superior alternative to conventional white-light endoscopy (WLE) for investigative purposes.

## Figures and Tables

**Table 1:** List of inclusion criteria.

Inclusion Criteria for Selected Articles in Review
Research that elucidated the diagnostic accuracy of narrow-band imaging (NBI) in the assessment of patients presenting with suspected laryngeal lesions.
Diagnostic parameters, including sensitivity and specificity.
Transparent specification of selection criteria.
Studies included were limited to those published in English.
The gold standard for diagnosing the lesion was established through histopathological examination.
Diagnostic methods included narrow-band imaging (NBI).

**Table 2:** List of exclusion criteria.

Exclusion Criteria for Selected Articles in Review
Suspicious lesions located outside the laryngeal region.
Reviews, editorials, commentaries, theses, and conference abstracts.
Complete extraction of data was not feasible.
Other diagnostic parameters except sensitivity and specificity.

**Table 3:** The characteristics of the studies included in the meta-analysis [22,23,24,25,26,27,28,29,30,31,32,33,34,35,36,37,38].

First Author	Publication Year	Patient No.	Study Type	Endoscopic System Used for NBI	Country	Pathological Examination Confirmed
Lin C et al. [22]	2021	112	Prospective	Olympus	China	77
Šatanková J et al. [23]	2021	589	Prospective	Olympus ENF-VH	Czech Republic	88
Popek B et al. [24]	2019	333	Prospective	Not Specified	Poland	129
Kraft M et al. [25]	2016	205	Prospective	Olympus ENF-VH	Switzerland	57
De Vito A et al. [26]	2017	158	Prospective	Not Specified	Italy	63
Sargunaraj JJE et al. [27]	2022	200	Prospective	Not Specified	India	48
Filipovsky T et al. [28]	2023	87	Prospective	Olympus Exera II	Czech Republic	13
Galli J et al. [29]	2021	196	Retrospective	Olympus	Italy	39
Watanabe A et al. [30]	2009	34	Prospective	Olympus ENF-V2	Japan	23
Bertino G et al. [31]	2015	248	Prospective	Olympus	Italy	15
Huang F et al. [32]	2017	57	Prospective	Olympus CV-190	China	14
Yang Y et al. [33]	2017	138	Prospective	Olympus ENF-VT2	China	57
Ni XG et al. [34]	2010	122	Prospective	Not Specified	China	73
Hagit Shoffel-Havakuk et al. [35]	2016	36	Prospective	Olympus	Israel	21
L. Stanıkova et al. [36]	2016	63	Prospective	Olympus	Berlin	22
Nikolaos Davaris et al. [37]	2018	163	Retrospective	Olympus	Germany	45
Robert Šifrer et al. [38]	2020	288	Prospective	Olympus	Slovenia	79

**Table 4:** Individual study findings obtained.

First Author (Year)	Sensitivity	Specificity
Popek B et al. (2019) [24]	0.985	0.6522
Robert Šifrer et al. (2020) [38]	0.98	0.947
Nikolaos Davaris et al. (2018) [37]	0.978	0.985
Bertino G et al. (2015) [31]	0.974	0.96
Kraft M et al. (2016) [25]	0.97	0.925
De Vito A et al. (2017) [26]	0.97	0.87
Galli J et al. (2021) [29]	0.95	0.9595
Lin C et al. (2021) [22]	0.9351	0.968
Ni XG et al. (2010) [34]	0.932	0.913
Watanabe A et al. (2009) [30]	0.916	0.846
Šatanková J et al. (2021) [23]	0.908	0.9556
L. Stanı’kova et al. (2016) [36]	0.88	0.956
Filipovsky T et al. (2023) [28]	0.7692	0.906
Yang Y et al. (2017) [33]	0.757	0.6119
Sargunaraj JJE et al. (2022) [27]	0.733	0.895
Huang F et al. (2017) [32]	0.697	1
Hagit Shoffel-Havakuk et al. (2016) [35]	0.5862	0.95

## Data Availability

All data are available by reference list of the article selected for this review.
